# Neural sensory stimulation does not interfere with the H-reflex in individuals with lower limb amputation

**DOI:** 10.3389/fnins.2023.1276308

**Published:** 2023-09-25

**Authors:** Suzhou Li, Ronald J. Triolo, Hamid Charkhkar

**Affiliations:** ^1^Department of Biomedical Engineering, Case Western Reserve University, Cleveland, OH, United States; ^2^Louis Stokes Cleveland Veteran Affairs Medical Center, Cleveland, OH, United States

**Keywords:** H-reflex, sensory restoration, lower limb amputation, neural interface, neuroprosthesis, plantar sensation

## Abstract

**Introduction:**

Individuals with lower limb loss experience an increased risk of falls partly due to the lack of sensory feedback from their missing foot. It is possible to restore plantar sensation perceived as originating from the missing foot by directly interfacing with the peripheral nerves remaining in the residual limb, which in turn has shown promise in improving gait and balance. However, it is yet unclear how these electrically elicited plantar sensation are integrated into the body’s natural sensorimotor control reflexes. Historically, the H-reflex has been used as a model for investigating sensorimotor control. Within the spinal cord, an array of inputs, including plantar cutaneous sensation, are integrated to produce inhibitory and excitatory effects on the H-reflex.

**Methods:**

In this study, we characterized the interplay between electrically elicited plantar sensations and this intrinsic reflex mechanism. Participants adopted postures mimicking specific phases of the gait cycle. During each posture, we electrically elicited plantar sensation, and subsequently the H-reflex was evoked both in the presence and absence of these sensations.

**Results:**

Our findings indicated that electrically elicited plantar sensations did not significantly alter the H-reflex excitability across any of the adopted postures.

**Conclusion:**

This suggests that individuals with lower limb loss can directly benefit from electrically elicited plantar sensation during walking without disrupting the existing sensory signaling pathways that modulate reflex responses.

## 1. Introduction

Over 50% of individuals with lower limb loss experience a fall within a year and report avoiding day-to-day activities because of their fear of falling (Miller et al., 2001). Among the highest priority needs desired by individuals with lower limb loss are to improve their ability to walk safely, to move around more independently, and to integrate back into the community without feeling excluded (Manz et al., 2022). Lack of sensory feedback from a missing foot could contribute to the observed deficits in gait stability and lower confidence in prosthesis use (Kim et al., 2023; Schmitt et al., 2023).

Plantar sensation contributes significantly to maintaining stability and avoiding falls while walking. It provides feedback about body position and how it is interacting with the environment, allowing us to respond appropriately to perturbations. A diminished plantar sensation, caused by desensitizing the foot’s plantar surface has been linked to decline in postural stability and compromised ability to recover from induced forward falls (Perry et al., 2000). Solutions to augment plantar sensation, such as the use of insoles with raised edges by the elderly, have demonstrated enhanced stability and balance, highlighting the role of plantar sensation in executing functional tasks (Maki et al., 1999).

Plantar sensation also contributes to the modulation of reflex pathways throughout the gait cycle, thereby promoting smooth and stable locomotion (Capaday and Stein, 1986; Zehr et al., 1997). These reflexes are selectively inhibited when the body prioritizes stability, while they are facilitated when reactive movements become a priority, such as obstacle clearance. This functional flexibility aligns with different recovery strategies employed by able-bodied individuals when experiencing perturbations at distinct stages of the gait cycle (Eng et al., 1994). Therefore, the integration of plantar sensation into our sensorimotor control schema underscores its significant role in maintaining stability during walking.

Individuals with lower limb loss can no longer rely on the plantar sensation from their missing feet. Consequently, they resort to different recovery strategies than able-bodied individuals, particularly when subjected to tripping incidents involving their intact limbs. The variety of movement patterns and stereotypical recovery strategies exhibited by individuals with lower limb loss are prioritized to establish the intact foot firmly on the ground to provide the sensory feedback and resulting confidence associated with knowledge of their body movement (Shirota et al., 2015). Nevertheless, the lack of natural sensory feedback from the prosthetic foot can still contribute to suboptimal recovery strategies and increased risk of falls.

Delivery of electrical stimulation to the remaining peripheral nerves in the residual limb after amputation can restore plantar somatosensation perceived as if arising from and co-located on the missing foot (Charkhkar et al., 2018; Petrini et al., 2019). Electrically elicited plantar sensation has been shown to address many user-identified needs associated with lower limb prostheses, such as improving gait symmetry and the perception of limb movement during gait (Kim et al., 2023), enhancing postural stability (Charkhkar et al., 2020), facilitating navigation through challenging terrains (Christie et al., 2020), and improving energy expenditure while walking (Petrini et al., 2019). Despite these reported functional benefits, it is still not fully understood how these electrically elicited plantar sensations are integrated with the retained spinal neural pathways, which are responsible for sensorimotor control. To investigate this, the present study employs the H-reflex to probe how these electrically elicited plantar sensations are processed by the spinal circuitry as compared to natural sensation from an intact foot.

The H-reflex serves as a simple model of sensorimotor control, and provides a tool to investigate how somatosensory inputs affect the modulation of reflex pathways (Misiaszek, 2003; Palmieri et al., 2004). The H-reflex is triggered by electrical stimulation to excite the Ia afferent fibers of the peripheral nerve (i.e., tibial nerve for the soleus H-reflex), which carry sensory information about muscle stretch from muscle spindles. The afferent fibers carry the signal to the spinal cord, where they synapse directly with the motoneurons that correspond to the originating muscle of the Ia fibers. The signal reaches the respective muscle, where the response can be measured via electromyography (EMG). The H-reflex exhibits context dependent modulation that differs with various activities (i.e., sitting, standing, walking, and running) and displays phase-dependency during cyclic motions such as walking (Misiaszek, 2003). Ultimately, the mechanisms that lead to this modulation rely on either presynaptic control that limits the number of Ia fibers that deliver the signal into the synaptic connection, or postsynaptic control which determines how many of the motoneurons are excitable by the incoming signal.

Plantar cutaneous sensation is intimately linked with the modulation of the H-reflex, a feature that varies with lower limb joint angles and loadings. Depending on hip angle, plantar cutaneous stimulation either inhibits the soleus H-reflex during hip flexion or facilitates it during hip extension (Knikou and Rymer, 2002). Another study found that plantar sensation significantly affects presynaptic and reciprocal inhibition of the H-reflex involving type I fibers (Pierrot-Deseilligny et al., 1981; Iles, 1996). Thus, the interaction between plantar cutaneous sensations and group I muscle afferents play an important role in regulating the excitability of spinal reflex pathways.

In the present study, we investigated the contribution of electrically elicited plantar sensations in sensorimotor control for individuals with lower limb loss, and focused specifically on its impact over medial gastrocnemius H-reflex excitability. We hypothesized that electrically elicited plantar sensations are integrated with other sensory inputs in the H-reflex pathway, reflecting a similar process to the sensory integration observed in an intact foot from previous studies.

## 2. Methods

### 2.1. Participants

**Figure 1:**
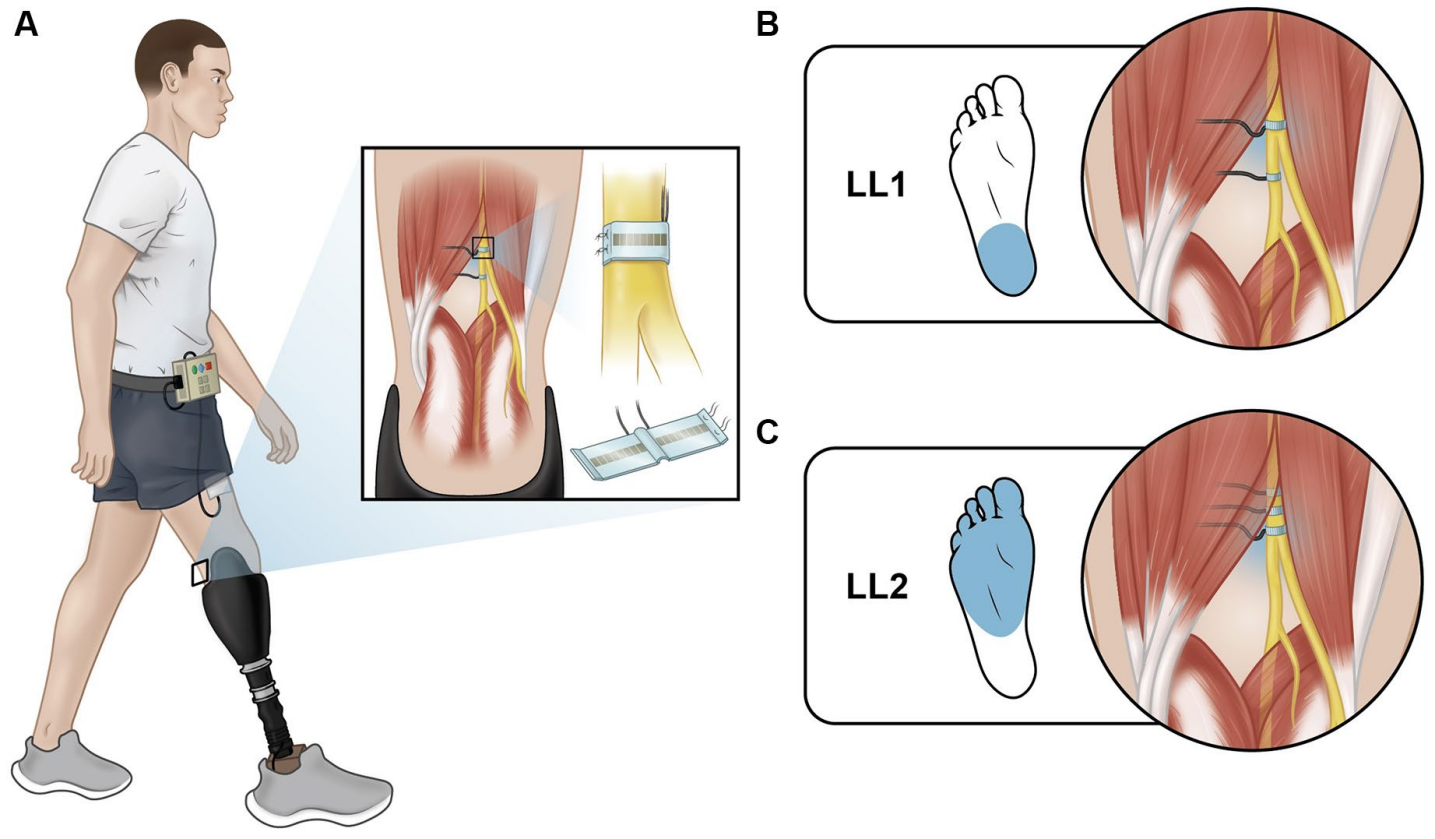
Electrically elicited plantar sensations (EPS). **(A)** Individuals with lower limb amputation were implanted with C-FINEs around their remaining peripheral nerves on their amputated side. Delivering electrical stimulation through these electrodes directly to the nerve elicited sensations that feel like they were coming from the individual’s missing foot. **(B)** LL1 had stimulation delivered through a contact on their tibial nerve and reported sensation from their heel. **(C)** LL2 had stimulation delivered through a contact on their sciatic nerve and reported sensation from their metatarsal and midfoot regions.

Two individuals with traumatic transtibial amputation participated in this study. At the time of enrollment, LL1 and LL2, aged 54 and 57 years old respectively, had each experienced limb loss nine years prior. Both participants were male, and they wore their clinically prescribed personal prostheses for all experiments. Neither participant had a medical history of neuropathy or peripheral vascular disease. Each had received 16-contact composite flat interface nerve cuff electrodes (C-FINEs) surgically installed around the remaining peripheral nerves in their residual limb above the knee (Figure 1A). LL1 had electrodes placed around their lower sciatic and tibial nerves (Figure 1B), and LL2 had electrodes located around their sciatic nerve (Figure 1C). The C-FINEs were connected to percutaneous leads exiting the body at the anterior mid-thigh to provide access to all available stimulating contacts. The details of the neural interface technology and the surgical procedure are described elsewhere (Charkhkar et al., 2018). LL1 had an internal ground within the cuff electrode, and LL2 had an external ground placed on the anterior superior iliac spine. At the time of this experiment, LL1 and LL2 had the system for six and three years, respectively. A custom-made and optically isolated external stimulator delivered current-controlled asymmetric charge-balanced cathodic waveforms to selected contacts within C-FINEs, which was controlled in real-time using a Speedgoat Real-Time Target Machine (Speedgoat, Inc.) via custom SIMULINK (MATLAB R2020b, Mathworks, Inc., MATLAB, 2020) programs. The system allowed independent control over pulse amplitudes (PAs) ranging from 0 to 5.6 mA, and pulse widths (PWs) varying from 0 to 255 μs. All experimental procedures were approved by the Louis Stokes Cleveland Veterans Affairs Medical Center Institutional Review Board and conducted under an Investigation Device Exemption from the United States Food and Drug Administration (IDE G110043), which governed the use of the neural technology. The participants gave their written informed consent prior to any research-related activities, following relevant human subject protection guidelines and regulations.

### 2.2. Postures

**Figure 2:**
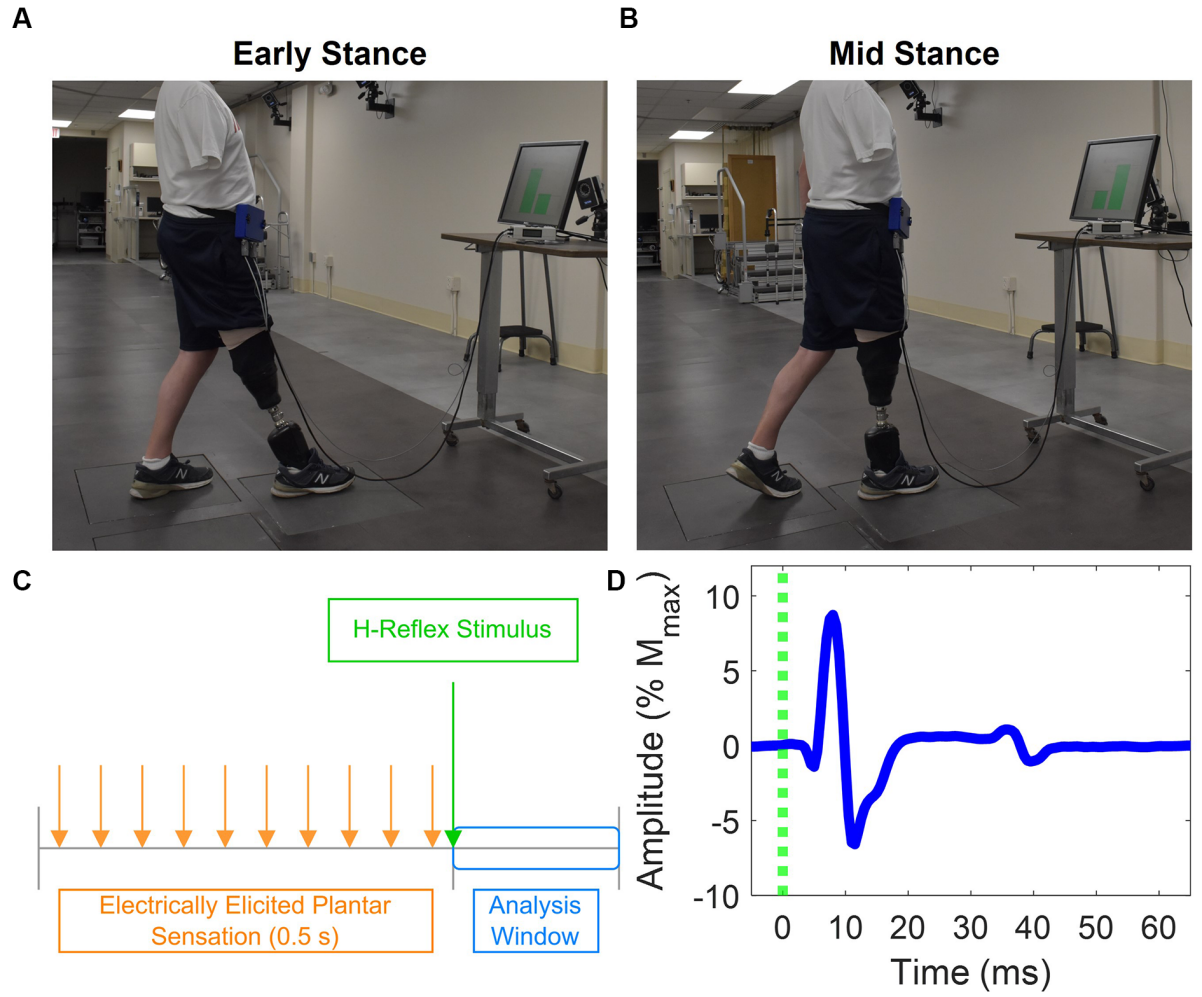
Experiment setup and H-reflex. Postures participants adopted that mimic **(A)** early stance and **(B)** mid stance. Each foot was placed on a force plate, and a computer monitor provided feedback about how much force was applied on each foot as a percentage of the participants’ body weight. **(C)** Stimulation paradigm used to evoke the H-reflex and provide EPS. EPS was delivered in a 0.5 s pulse train. The H-reflex stimulus was delivered 30 ms after the last pulse of the EPS pulse train. EMG is analyzed in a small window after delivery of the H-reflex stimulus. **(D)** Representative EMG showing medial gastrocnemius H-reflex response. The M-wave and H-reflex were identified between 0 to 20 ms and 30 to 50 ms, respectively.

Participants assumed one of two postures to simulate different phases of the gait cycle: early stance (Figure 2A) and mid-stance (Figure 2B). They placed their prosthetic foot in front of their intact foot, with each foot resting on a separate force plate (Advanced Mechanical Technology, Inc.). Prior to starting the experiments, they stood still on each individual force plate to capture body weight (BW) for normalization purposes. The early stance and mid-stance postures were simulated by maintaining a load of 30% ± 5% and 70% ± 5% of their BW on the leading prosthetic limb, respectively. To achieve the desired posture, participants were instructed to shift their weight onto their prosthetic limb as if they were going to take a step until they reached the target load. Participants received visual feedback via a computer monitor displaying bar plots indicating the extent to which their limbs were loaded (Figures 2A,B). The bars turned green when the loading was within the target range and red when it was outside the intended range. Prior to each data collection session, participants practiced these postures until they could comfortably achieve them and hold the target loads. During the experiment, once the target load was attained, participants maintained the posture until instructed to return to restful standing with feet under the pelvis and shoulder width apart. Participants lightly touched the back of a chair with two fingers at all times irrespective of whether it was perceived as necessary to maintain balance.

Each test posture was maintained for up to one minute, during which the H-reflex was evoked four to five times. During the experiment, participants were instructed to take a seated break about every 20 minutes or whenever they requested one.

### 2.3. Electrically elicited plantar sensation

Stimulation was delivered by different contacts within the electrodes around the tibial nerve of LL1 and sciatic nerve of LL2 (Figure 1) to elicit plantar sensations and to evoke the H-reflex. Plantar sensation was always elicited by a contact that was not adjacent to the one utilized for the H-reflex (see section 2.4 for details on evoking the H-reflex). To provide elicited plantar sensation (EPS), stimulation with an inter-pulse interval (IPI) of 50 ms lasting for a duration of 0.5 s was applied prior to delivery of the H-reflex stimulus. The perceived intensity of EPS was modulated to align with the two levels of force applied to the prosthetic foot on the leading force plate. The PA was held constant, and the PW was varied between two pre-determined levels. Stimulation parameters were determined at the start of the first session for each participant (Table 1). Participants reported perceived intensity levels based on an individual open-ended self-selected scale. This participant-specific scale was established by each participant, where a score of 0 indicated no sensation, and any other number was chosen based on the intensity of the sensation perceived during the initial trial. Subsequent trials were rated according to this initial benchmark, offering a flexible scale without an upper limit (Graczyk et al., 2016). During the early stance posture, when the prosthetic limb was loaded to 30% BW, stimulation parameters were adjusted such that the EPS intensity was slightly over sensory threshold. A PA was selected based on the amplitudes utilized during functional use of EPS during gait, and the PW was increased until the participant reported feeling the sensation. In the mid-stance posture, wherein the prosthetic limb was loaded to 70% BW, the same PA was used as in early stance, and the PW was increased until the reported intensity sensation was approximately double that experienced during the early stance posture. This ensured proportional sensory feedback reflective of the limb loading.

**Table 1:** Electrical stimulation parameters for delivering electric current through the implanted C-FINEs to elicit EPS and evoke H-reflex.

Participant	LL1		LL2	
**Electrically elicited plantar sensation (EPS)**				
	*Early stance*	*Mid stance*	*Early stance*	*Mid stance*
C-FINE contact	13		16	
PA (mA)	1.0		0.9	
PW (μs)	80	120	100	120
Charge density (μC/cm^2^)	15.9	23.9	17.9	21.5
IPI (ms)	50			
**H-reflex stimulus**				
C-FINE contact	2		10	
PA (mA)	1.0–4.0		1.0–2.6	
PW (μs)	100		100	
Charge density (μC/cm^2^)	19.9–79.6		19.9–51.7	

### 2.4. H-reflex

The H-reflex stimulus was a single pulse delivered 30 ms after the last pulse from the EPS train (Figure 2C) through a contact that had been identified prior to the start of the experiment to evoke a consistent H-reflex response. Stimuli were randomly delivered at intervals 8 to 10 s apart to avoid refractory effects from the preceding stimulus. To obtain H-reflex recruitment curves, the stimulus PW was held constant at 100 μs, while the PA was randomly increased until the maximum M-wave was observed and decreased until the H-reflex disappeared. Stimulation parameters to evoke the H-reflex for each participant are shown in Table 1.

### 2.5. Electromyography

Wearable electromyography (EMG) sensors (Delsys, Inc.) captured M-wave and H-reflex response from the medial gastrocnemius muscles in the residual limbs. Signals were sampled at 1,926 Hz. For H-reflex measurements, the medial gastrocnemius muscle was identified by palpating the residual limb while the participants were instructed to point the toes of their missing foot downward (plantarflex). The EMG sensor was positioned where the contraction was found to be most significant. This site was subsequently confirmed by confirming EMG activation during plantarflexion and ensuring there was no activation during dorsiflexion of the missing foot.

### 2.6. Data analysis and outcome metrics

Data were analyzed in MATLAB (R2020b, Mathworks Inc., MATLAB, 2020) and R Statistical Software (v 4.2.2, R Core Team, 2022). In each trial, the load applied to each limb was quantified by measuring the average force magnitude from a time window spanning from 1 s before H-reflex stimulus delivery to 100 ms after the delivery.

EMG signals were digitally filtered between 20 and 450 Hz by a 4th order zero-phase Butterworth filter, and 60 Hz power line noise was removed using a spectrum estimation technique (Mewett et al., 2001; Eiber, 2015). M-waves and H-reflexes were identified within specific time windows following the delivery of the H-reflex stimulus of 0 to 20 ms and 30 to 50 ms, respectively (Figure 2D). To remove M-wave contamination in the H-reflex window, a template of the M-wave form was generated by averaging the EMGs for trials where the maximum M-wave was evoked. For each trial, this template was proportionally adjusted to match the M-wave amplitude of that trial, and the resulting template was then subtracted from the respective trial’s EMG. This is consistent with previously reported methodology to minimize the M-wave effect from quadriceps H-reflexes (Larsen et al., 2006).

While a single stimulus typically resulted in a dominant H-reflex morphology across trials, some trials exhibited no H-reflex response, and others had sporadic muscle responses that did not align with the primary H-reflex morphology. As such, we employed two additional criteria followed by a principal component analysis (PCA) to ensure only the primary H-reflex response was included in the analysis. First, only trials were considered for which both the detected M-wave and H-reflex amplitudes exceeded 1% of the maximum M-wave amplitude. This was to confirm the presence of a M-wave in response to the stimulus (Palmieri et al., 2004). Secondly, detected waveforms had to be above the background EMG levels. Trials were only included if the detected M-wave and H-reflex amplitudes surpassed four times the standard deviation of the EMG between 600 to 700 ms prior to delivery of the H-reflex stimulus.

Subsequently, the PCA identified the primary H-reflex response among the remaining trials. The template M-wave was subtracted from each trial, and data was extracted and normalized by the range of the signals within the H-wave window prior to identifying the principal components. Hierarchical clustering was performed on the first two principal components to identify the cluster containing the primary H-reflex. The peak-to-peak amplitudes of the M-waves and primary H-reflexes were calculated for each trial and normalized by the maximum M-wave for each experimental condition.

Lastly, we examined the background EMG to gauge the excitability of the motoneuron pool, an important parameter influencing H-reflex amplitude (Misiaszek, 2003; Palmieri et al., 2004). The background EMG was quantified by taking the root mean square of the EMG within a span of 600 to 700 ms preceding delivery of the H-reflex stimulus. This background EMG measurement was then normalized by the maximum M-wave amplitude.

### 2.7. Statistical analysis

Data were analyzed separately for each participant. For all statistical analyses, the significance level was set to 0.05.

To determine if the postures were maintained consistently throughout the experiment, a type III analysis of variance (ANOVA) was conducted to compare the magnitude of the load on the prosthetic limb (as % BW) for the EPS and the posture conditions. A *post-hoc* Tukey test for multiple comparisons identified if there was a difference in loading between EPS conditions for each posture.

Linear regression models were generated to determine how posture and EPS conditions affect the ascending portion of the H-reflex recruitment curve. In this model, the predictors for the normalized H-reflex amplitude were the normalized M-wave amplitude, EPS conditions, posture conditions, and their interactions. A type III sequential ANOVA on the linear regression model determined which predictors significantly affected the H-wave amplitude.

Another measure of reflex excitability was the ratio between the maximum H-reflex amplitude (H_max_) and the maximum M-wave amplitude (M_max_). The H_max_ was derived from the H-reflex data for which the M-wave amplitudes were the highest 10% observed during the ascending limb of the H-reflex recruitment curve. A type III ANOVA was used to determine the effect of posture and EPS on the H_max_ to M_max_ (H_max_/M_max_) ratio.

Additionally, a type III ANOVA was performed on the normalized background EMG magnitudes with EPS and posture conditions as factors. A *post-hoc* Tukey test for multiple comparisons was performed to determine if the average background EMG magnitudes differed between EPS conditions during specific postures.

The H-reflex gain over the background EMG was characterized for postures with a significant difference in background EMG between the EPS conditions. For each posture, a separate linear regression model was generated with the normalized background EMG magnitude, EPS conditions, and their interaction as predictors for the normalized H-reflex amplitude. A sequential type III ANOVA on the linear regression model determined if EPS affected the relationship between the background EMG magnitude and H-reflex amplitude.

## 3. Results

### 3.1. EPS locations and intensities

Both participants reported perceiving the EPS as originating from their missing foot. Notably, the perceived intensity during mid-stance was higher compared to early stance. LL1 located the sensation at the heel (Figure 1B), reporting intensity levels of 0.3 and 0.5 on their participant-specific scale during early and mid-stance postures, respectively. LL2 perceived the EPS in the metatarsal and midfoot regions (Figure 1C). LL2’s reported perceived intensities in ranges of 0.5 to 2 and 1 to 3 for early and mid-stance postures, respectively. After determining the initial stimulation parameters in the two postures, the stimulation parameters were held constant because previous work has shown that sensory detection thresholds typically remain constant (Christie et al., 2019). Both participants reported feeling a tingling sensation from their described locations.

### 3.2. Maintaining postures

Participants successfully achieved and maintained the specified postures for the duration of the experiment with a consistent level of limb loading for early and mid-stance positions across stimulation conditions. For LL1, the average load of the leading limb during the early stance posture was 31.5% ± 0.1% BW without EPS and 32.0% ± 0.2% BW with EPS. Conversely, the average load on the leading limb was 70.7% ± 0.1% without EPS and 70.7% ± 0.1% BW with EPS during the mid-stance posture. The ANOVA indicated significant primary effects from posture (*p* < 0.001) and EPS (*p* = 0.02), as well as a significant interaction between the two (*p* = 0.049). The *post-hoc* test identified no significant differences in loading between EPS conditions for both the early stance (*p* = 0.09) and mid-stance (*p* = 1.00) postures. For LL2, the average load of the leading limb across the trails was 30.1% ± 0.1% BW without EPS and 30.0% ± 0.2% BW with EPS for the early stance posture. In the mid-stance posture, their average leading limb load across the trials was 71.5% ± 0.2% BW without EPS and 71.4% ± 0.2% BW with EPS. As expected, posture had a significant influence on loading (*p* < 0.001), and no significant differences were observed on loading from EPS (*p* = 0.99) or interaction of EPS with posture (*p* = 0.89). These results confirmed that the participants consistently achieved and maintained their postures and desired loading conditions to simulate gait.

### 3.3. Effects of posture and EPS on the H-reflex

**Figure 3:**
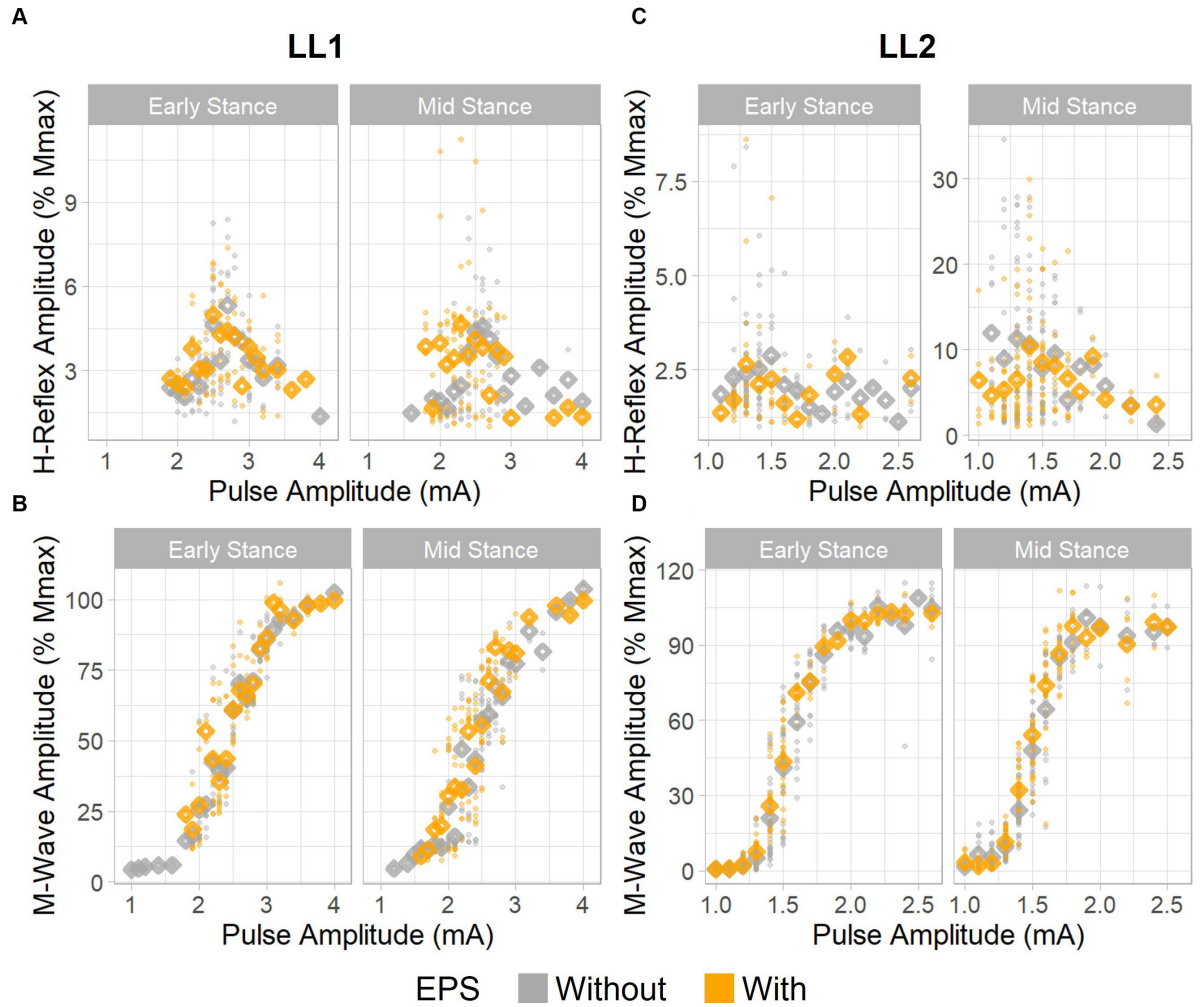
H-reflex and M-wave recruitment curves. H-reflex recruitment curves for **(A)** LL1 and **(C)** LL2. M-wave recruitment curves for **(B)** LL1 and **(D)** LL2. Individual data points are denoted as small circles, whereas the mean values per pulse amplitude level are represented as diamonds.

**Figure 4:**
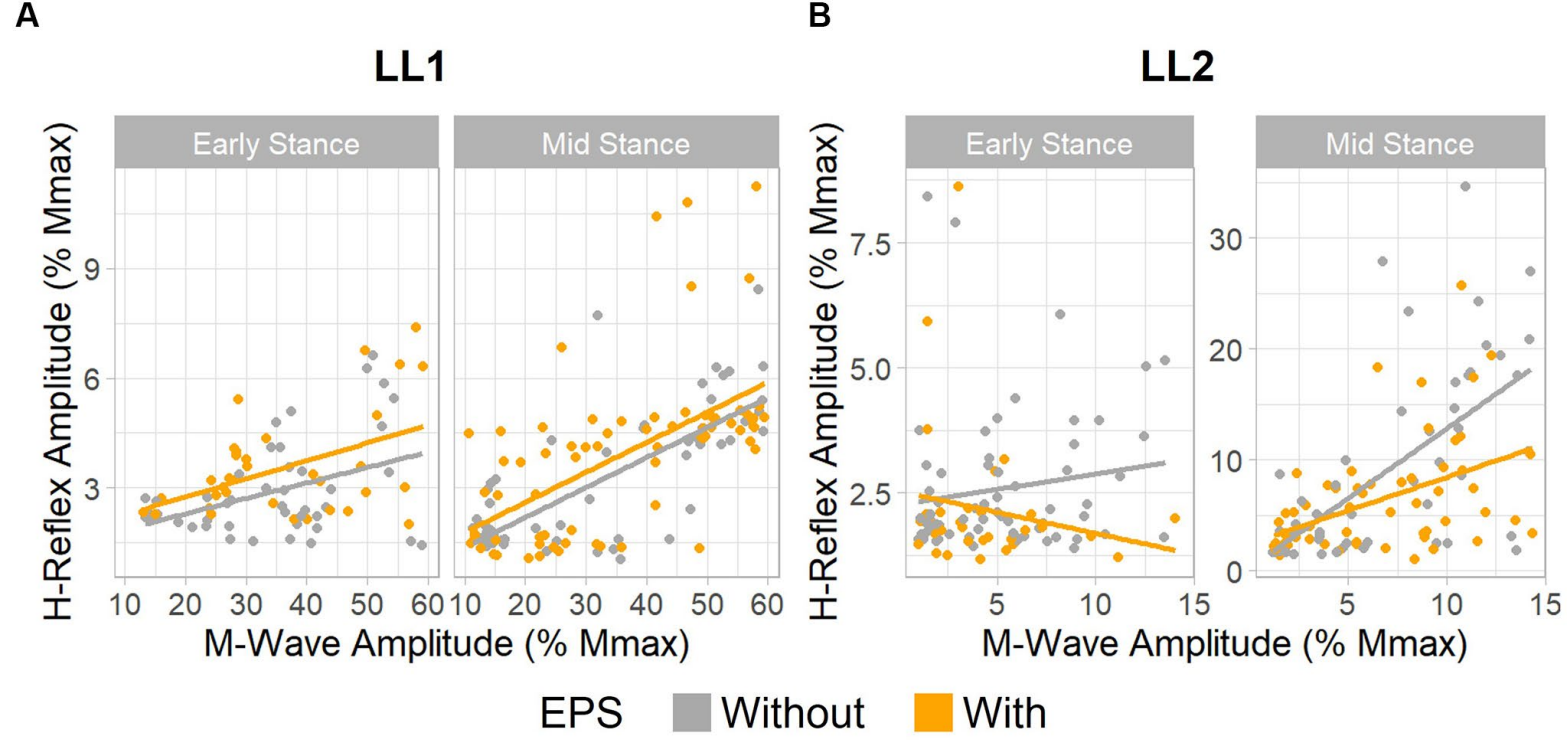
EPS does not significantly influence H-reflex amplitudes. Relationship between H-reflex amplitude and stimulus intensity, as measured by M-wave amplitude. **(A)** Data for LL1 on the ascending limb of the H-reflex recruitment curve existed between 10% and 60% the maximum M-wave amplitude [early stance: without EPS (*n* = 45) and with EPS (*n* = 34), mid-stance: without EPS (*n* = 60) and with EPS (*n* = 71)]. **(B)** Data for LL2 on the ascending limb of the H-reflex recruitment curve was found under 15% the maximum M-wave amplitude [early stance: without EPS (*n* = 65) and with EPS (*n* = 33), mid-stance: without EPS (*n* = 53) and with EPS (*n* = 59)].

For LL1, the full H-reflex and M-wave recruitment curves are illustrated in Figures 3A,B, respectively. H-reflexes on the ascending portion of the H-reflex recruitment curve were found within a M-wave amplitude range of 10%–60% of the maximum (Figure 4A) for the early stance posture without and with EPS (*n* = 45 and *n* = 34, respectively), and mid-stance without and with EPS (*n* = 60 and *n* = 71, respectively). The regression model demonstrated a significant correlation of the predictors with the H-reflex amplitude (*p* < 0.001, R^2^ = 0.40). The sequential ANOVA revealed that only M-wave amplitude (*p* = 0.02) had a significant effect on the H-reflex amplitude. Posture (*p* = 0.25) and the interaction between M-wave amplitude and posture (*p* = 0.06) had no effect on the H-reflex amplitude. Additionally, EPS (*p* = 0.74), the interaction of EPS with M-wave amplitude (*p* = 0.79), the interaction of EPS with posture (*p* = 0.94), and the interaction between all three predictors (*p* = 0.82) had no significant effect on the H-reflex amplitude. Table 2 delineates the H_max_/M_max_ ratios for LL1. The ANOVA determined that posture (*p* = 0.09), EPS (*p* = 0.37), and the interaction between them (*p* = 0.39) did not significantly influence the H_max_/M_max_ ratio.

**Table 2:** H_max_ to M_max_ ratios for both participants as a function of posture and application of EPS.

		Electrically elicited plantar sensation (EPS)	
		*Without*	*With*
		**LL1**	
Posture	*Early stance*	4.1% ± 2.1% (*n* = 7)	5.0% ± 2.1% (*n* = 6)
	*Mid stance*	5.5% ± 1.1% (*n* = 14)	5.4% ± 1.8% (*n* = 17)
		**LL2**	
	*Early stance*	2.7% ± 1.2% (*n* = 32)	1.8% ± 0.6% (*n* = 10)
	*Mid stance*	13.3% ± 9.1% (*n* = 29)	8.0% ± 5.8% (*n* = 35)

The H-reflex and M-wave recruitment curves for LL2 are shown in Figures 3C,D, respectively. Early and mid-stance H-reflexes for LL2 were predominantly present for M-waves less than 15% of the maximum M-wave amplitude (Figure 4B). Based on these observations, H-reflexes were analyzed within this window for early stance without and with EPS (*n* = 65 and *n* = 33, respectively), and mid-stance without and with EPS (*n* = 52 and *n* = 59, respectively). A significant relationship of the predictors on the H-reflex amplitude was found through the linear regression model (*p* < 0.001, R^2^ = 0.48). The sequential ANOVA determined only a significant effect of the interaction between M-wave amplitude and posture (*p* < 0.001). However, M-wave amplitude (*p* = 0.68) and posture (*p* = 0.18) independently had no significant effects. Consistent with findings from LL1, there were no significant effects of EPS (*p* = 0.86), the interaction of EPS with M-wave amplitude (*p* = 0.61), the interaction of EPS with posture (*p* = 0.39), and the interaction between all three predictors (*p* = 0.15). LL2’s H_max_/M_max_ ratios are presented in Table 2. Consistent with the linear regression findings, only posture exhibited a significant effect on the H_max_/M_max_ ratio (*p* < 0.001). Conversely, EPS (*p* = 0.68) and the interaction between EPS and posture (*p* = 0.09) had no significant effects on the H_max_/M_max_ ratio.

These observations indicate that EPS had no influence on the amplitude of the H-reflex amplitude for either participant. Only LL2 exhibited an influence of posture over the H-reflex amplitude.

### 3.4. Effects of EPS on gain of the H-reflex over background EMG

**Figure 5:**
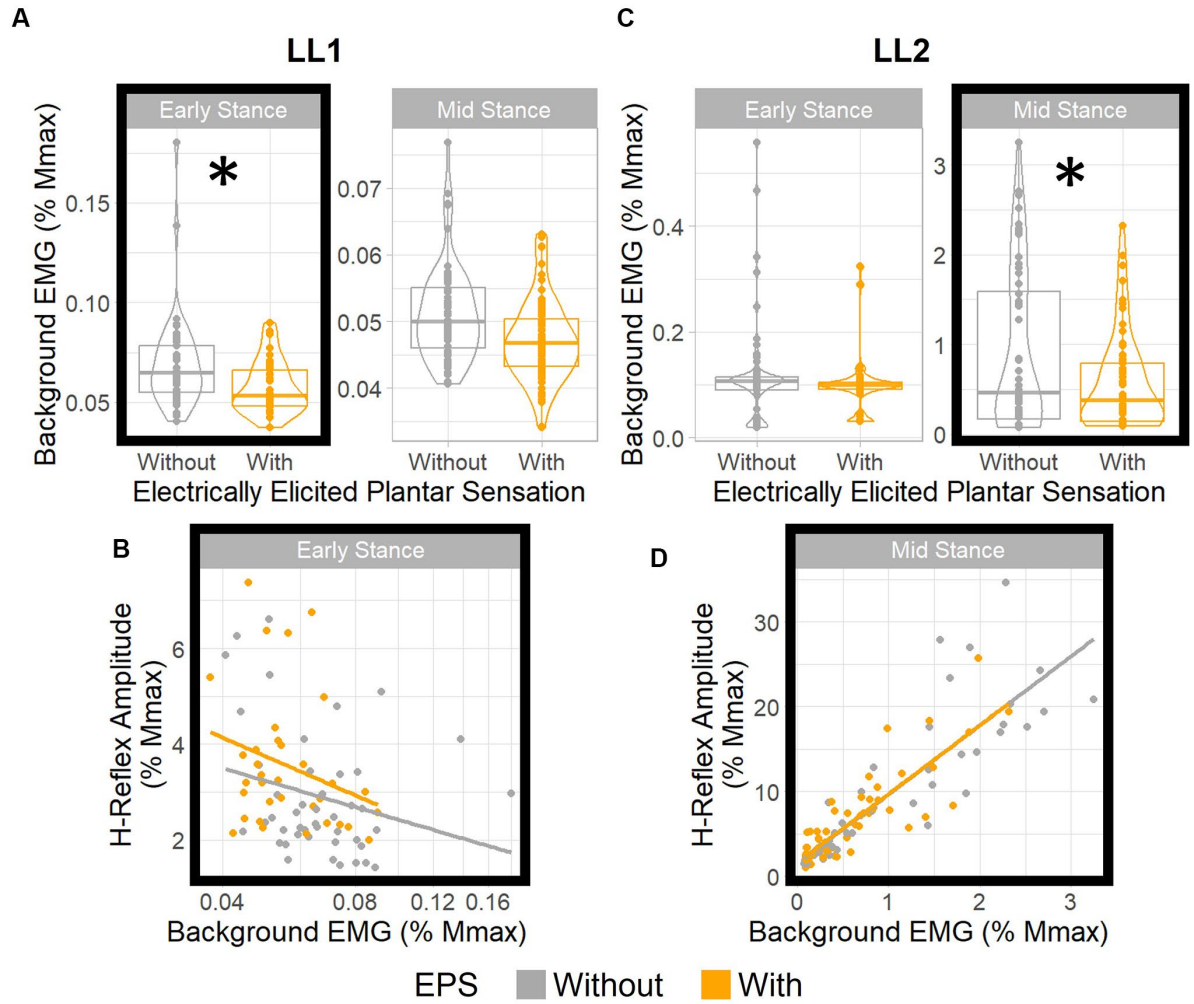
EPS does not significantly influence H-reflex gain. Boxplots show distributions of background EMG for **(A)** LL1 and **(C)** LL2 for different EPS and posture conditions. * denotes statistically significant differences in the background EMGs between the EPS conditions in each posture. H-reflex amplitude vs. background EMG **(B)** for LL1 (adjusted to a log10 scale) in early stance and **(D)** for LL2 in mid stance. For both participants, EPS had no effect on the relationship between the background EMG and the H-reflex amplitude.

For LL1, posture (*p* < 0.001) and EPS conditions (*p* < 0.001) had significant effects on the background EMG magnitude (Figure 5A), but the interaction between the two factors did not (*p* = 0.052). The *post-hoc* test demonstrated a marked EPS effect on background EMG magnitude in early stance (*p* = 0.001) but not in the mid-stance posture (*p* = 0.32). Consequently, we investigated the effect of the background EMG on the H-reflex excitability during early stance for this participant via regression analysis. Because the data were skewed toward smaller values, a logarithmic transform was applied to the background EMG magnitude, resulting in a regression model that fit the data (*p* = 0.027, R^2^ = 0.11; Figure 5B). The EPS appeared to have no significant effect on the relationship between background EMG and H-reflex amplitude. This was supported by the sequential ANOVA which revealed no significant impact from the background EMG, EPS, or their interactions (*p* = 0.1, *p* = 0.74, and *p* = 0.66, respectively) on the H-reflex amplitude. This finding indicates that consistent reflex gain was maintained despite differences in motoneuron excitability between the EPS conditions during early stance.

For LL2, posture (*p* < 0.001) and the interaction between posture and EPS (*p* = 0.03) had significant effects on the background EMG magnitude, but EPS independently had no effect (*p* = 0.91; Figure 5C). The *post-hoc* test indicated that the background EMG magnitude was significantly different between EPS conditions in the mid-stance posture (*p* = 0.004) and not in the early stance posture (*p* = 1.00), which differed from what was observed with LL1. Subsequent analysis with a linear model (*p* < 0.001, R^2^ = 0.76) corroborated that H-reflex gain was constant (Figure 5D). Although there was a significant effect of background EMG on the H-reflex amplitude (*p* < 0.001), EPS had no significant effect (*p* = 0.89), nor was there an interaction between background EMG and EPS (*p* = 0.95). Consequently, we deduced that EPS did not affect the relationship between background EMG and H-reflex amplitudes. As such, for both participants, EPS had no effect on reflex gain regardless of variations in motoneuron excitability.

## 4. Discussion

Our findings reveal that EPS does not disrupt spinal reflex pathways in static postures. Contrary to our original hypothesis, EPS did not alter the H-reflex amplitudes as shown by the consistency between the H-reflex and M-wave relationship along the ascending portion of the H-reflex recruitment curve, and the consistency of the H_max_/M_max_ ratios. While certain postures exhibited different levels of motoneuron excitability across EPS conditions, our analysis identified that EPS did not influence the H-reflex gain relative to the background EMG magnitude. Nevertheless, the existing natural sensory inputs in individuals with lower limb loss can still modulate reflex pathways, as evidenced by differences in the H-reflex amplitudes between the different postures for one participant. Combined, these observations indicate EPS does not affect the overall reflex excitability, and there is a clear interplay between the available sensory inputs from the joints and muscles.

Reflex excitability is modulated over the gait cycle based on the body’s need to prioritize stability. Our participants were instructed to adopt postures that mimicked early stance and mid-stance phases of the gait cycle. For able bodied individuals, the soleus H-reflex is typically inhibited in early stance because the body prioritizes stability during weight acceptance. Contraction of the triceps surae muscles during this phase would lead to plantarflexion, which would be counter-productive to stable loading of the limb. As gait proceeds toward mid or late stance, the soleus H-reflex amplitude increases since plantarflexion could facilitate push-off as the gait cycle enters the propulsive phase (Capaday and Stein, 1986). This modulation is made possible by the different sensory inputs entering the spinal cord during gait and the descending inhibition or facilitation of the neural circuitry that controls locomotion. Even when adopting static postures mimicking gait, different sensory inputs related to joint angles, limb loads, and plantar pressure distributions are processed by the spinal cord. And, these inputs can still contribute to modulation of H-reflex excitability (Fung and Barbeau, 1994). Our participants exhibited some hip flexion on their amputated side, which induces a stretch in the hamstrings. In the mid-stance posture, the participants exhibited less hip flexion and increased load at the knee joint when compared to early stance. Additionally, internal strategies that participants use to adopt these postures will activate different muscles, therefore contributing different mechanoreceptor inputs to the spinal cord. We observed that the medial gastrocnemius H-reflex was influenced by different postures in one participant. This indicates that the existing natural sensory inputs, as mentioned above, can still contribute to reflex modulation.

H-reflex excitability is a result of multiple inputs from the body contributing different inhibitory and excitatory effects on the H-reflex circuitry. Proprioceptive signals from type I fibers are known to be some of the main contributors to modulating the H-reflex during gait (Misiaszek, 2003; Kamibayashi et al., 2010). These proprioceptive fibers include type Ia fibers that carry information about muscle stretch, and type Ib fibers that carry information about muscle tension. Natural plantar cutaneous sensation, which originates from skin mechanoreceptors on the plantar surface of an intact foot, can modulate the inhibitory mechanisms of these type I fibers. Type Ia fibers can exert direct control on the H-reflex arc through presynaptic and postsynaptic mechanisms. Stimulation of cutaneous afferents in the feet can reduce the level of presynaptic inhibition from stimulation of antagonist Ia fibers on the soleus H-reflex (Iles, 1996). Simultaneously, it also increases the effect of reciprocal inhibition on the ipsilateral soleus H-reflex and depresses this effect on the contralateral soleus H-reflex (Rossi and Mazzocchio, 1988). Furthermore, it has been shown that the effect of plantar cutaneous sensation on H-reflex excitability depends on hip angle, where Ia fibers can carry information about the stretching of muscles controlling the hip (Knikou and Rymer, 2002). One of the primary mechanisms type Ib fibers inhibit the H-reflex is through autogenic inhibition, where increased muscle tension inhibits the motoneuron pool. Natural plantar cutaneous sensation depresses Ib inhibition in muscles controlling the knee. However, it has been shown to have no effect on autogenic Ib inhibition of the triceps surae, because autogenic inhibition is important in allowing these muscles to contract and plantarflex the foot while they are lengthening under dorsiflexion during the stance phase (Pierrot-Deseilligny et al., 1981). There are also other inputs to the spinal circuitry outside the type I fibers that can modulate H-reflex excitability. Knee and ankle joint-load signals can inhibit the H-reflex (Nakazawa et al., 2004), and plantar cutaneous sensation can modulate descending control of H-reflex excitability (Kamibayashi et al., 2010). Previous studies with able-bodied individuals had shown that natural plantar cutaneous sensation elicited by electrically stimulating the mechanoreceptors on the plantar surface of the foot integrated with other inputs, as mentioned above, to inhibit the H-reflex in different postures that mimicked walking (Fung and Barbeau, 1994). As we found that EPS does not affect H-reflex amplitude under varying postures, it suggests that activation of cutaneous afferents via electrical stimulation does not alter proprioceptive and descending circuitry in the reflex pathways, at least during this static limb loading task.

In our study, EPS may not have significantly affected H-reflex excitability due to compromised inputs to the spinal cord. Our participants lack an ankle joint and associated muscular control below the knee. The process of repositioning and securing the residual muscles during amputation surgery varies on a case-by-case basis. As a result, the stretch and tension experienced by these muscles may diverge from that of able-bodied individuals (Fung and Barbeau, 1994). Moreover, existing evidence suggest reorganization in cortical motor areas corresponding to amputated limbs is common among individuals with limb loss, which could potentially alter the descending input into the spinal cord (Chen et al., 1998). Such compromised inputs could possibly explain why LL1 did not exhibit any effects of posture on their H-reflex amplitude. For LL2, however, a combination of sensory inputs from the residual muscles and from the rest of the body, such as from the muscles controlling the hip and the knee, can be contributing to modulation of H-reflex excitability between postures. Yet, for both participants, the original inhibitory mechanisms, especially those involving type I fibers in the triceps surae, may no longer be intact. Therefore, EPS may be interacting with the control mechanisms originating from these compromised sources and leading to an apparent lack of modulation in H-reflex pathways by EPS.

The findings of our study reinforce the intricate nature of reflex modulation, and static postures may not directly translate to the dynamics in complex tasks such as walking. While our study simulated two key instances of the gait cycle, a comprehensive assessment of H-reflex during actual walking might reveal different interactions with EPS. Even in individuals with lower limb loss, the residual limb muscles often exhibit periodic activation during walking, at times resembling patterns observed in able-bodied individuals (Huang and Ferris, 2012). However, considering the unique repositioning and attachment of muscles in each amputation surgery, correlating these activation patterns directly with those of able-bodied individuals remains challenging. Furthermore, descending inputs into the spinal cord during gait could add another layer of complexity to H-reflex modulation. While our study provides initial insights into the modulation of the H-reflex by EPS, further investigations will seek to elucidate the role of EPS in more complex spinal reflex pathways during dynamic activities such as walking.

The type of nerve fibers activated by EPS could not be precisely controlled. Previous studies elicited plantar cutaneous sensation by electrically stimulating the skin on the plantar surface of the foot or the cutaneous nerves innervating the foot through the surface of the skin. In this study, EPS was produced by directly and selectively stimulating the nerve to activate the fibers that are hypothesized to have once connected to these cutaneous mechanoreceptors pre-amputation. This is supported by our participants consistently reporting feeling sensations perceived as arising from their missing feet with EPS, which suggests that EPS likely activated cutaneous fibers. However, electrical stimulation of the nerve may also activate other proprioceptive fibers. This could send a mix of inhibitory and excitatory signals to the spinal cord, potentially resulting in no noticeable changes in H-reflex excitability. Additionally, the activation pattern received by the spinal cord via EPS might not mirror those from natural activation of plantar cutaneous mechanoreceptors. Hence, these distinct afferent encodings may be interpreted and processed differently within the spinal reflex pathways.

In this work, we employed a unique methodology to evoke the H-reflex, differing from traditional methods typically seen in the literature. In conventional studies, the H-reflex is evoked via transcutaneous electrical stimulation of the tibial nerve at the popliteal fossa (Misiaszek, 2003; Palmieri et al., 2004). This technique non-selectively stimulates the entire tibial nerve. In contrast, we delivered stimulation to the nerve through a single contact on an implanted high-density nerve cuff electrode, which allows for spatially selective activation of nerve fibers. As mentioned, different contacts were tested to identify the one that most consistently recruited type Ia fibers into the H-reflex arc. However, this stimulation inherently targets a subset of type Ia fibers, unlike the broader recruitment seen in classical studies. This could explain the lower H_max_/M_max_ ratios presented in our study compared to earlier reports. Previous studies have demonstrated that elderly individuals consistently show lower H_max_/M_max_ ratios for the soleus H-reflex when in a supine position compared to younger individuals (Koceja and Mynark, 2000; Kido et al., 2004; Tsuruike et al., 2012). This observation provides insight into how small H_max_/M_max_ ratios may affect the modulatory effects on the H-reflex. It has been suggested that the lower H_max_/M_max_ ratios in the elderly may be influenced by changes in the presynaptic or postsynaptic modulatory mechanisms on the H-reflex arc, or by a decrease in the Ia inputs to the arc (Kido et al., 2004). Similarly, with our peripheral nerve stimulation approach, it is possible that the activation of only a subset of Ia fibers entering the H-reflex arc could influence the H_max_/M_max_ ratio. Furthermore, it has been shown that heteronymous conditioning by stimulation of the femoral nerve has a smaller effect on the H-reflex in elderly populations when compared with younger individuals, indicating sensory inputs contribute differently to modulatory mechanisms present in individuals with smaller H_max_/M_max_ ratios (Koceja and Mynark, 2000; Tsuruike et al., 2012). Even considering the demographic of our participants, which closely mirrors the elderly groups in prior studies, the H_max_/M_max_ ratios we observed are still smaller than those reported within the literature. Evidence from these studies suggests that individuals with smaller H_max_/M_max_ ratios have different modulatory mechanisms controlling the H-reflex arc than those with larger ratios. This strengthens our assertion that the smaller Ia inputs into the H-reflex arc might have contributed to the H_max_/M_max_ ratios observed in our study. To our knowledge, there are no comprehensive examinations of how selective nerve stimulation affects the H-reflex response and its interactions with other somatosensory pathways. Our study indicates that activation of a subset of fibers into the H-reflex arc, which produces a small H_max_/M_max_ ratio, may not be significantly affected by inhibitory mechanisms.

We precisely controlled the interval timing between the end of the EPS and the test pulse to evoke the H-reflex. This interval was chosen to maximize the possible effect of cutaneous stimulation on the H-reflex in different postures based on previously reported values (Fung and Barbeau, 1994). In these prior studies, plantar cutaneous sensation was elicited via distal stimulation at the foot. In contrast, our approach involved the delivery of electrical stimulation via a nerve cuff electrode, located significantly more proximally than the foot. Despite the spatial difference and the conduction time needed to bridge the gap, we did not change the interval timing to ensure consistency. However, the timing by which the conditioning signal reaches the spinal cord prior to activation of the H-reflex is known to influence the effect of plantar cutaneous sensation on the spinal reflex pathways (Fung and Barbeau, 1994). As an extension of this work, future studies could experiment with various timings to discern if there exists an interval where EPS can effectively modulate H-reflex excitability.

Multiple reports have shown advantages of restoring plantar sensation using peripheral neural interface technology. This restored sensation is essential for maintaining postural stability (Charkhkar et al., 2020) and navigation through challenging terrains (Christie et al., 2020). It improves gait symmetry and limb speed perception (Kim et al., 2023), and decreases energy expenditure during walking (Petrini et al., 2019). Additionally, the sensorimotor system processes the restored plantar sensation achieved through this EPS technology akin to natural sensation (Shell et al., 2021). Our findings suggest that individuals with lower limb loss can use this EPS technology to improve their functional outcomes without any negative interference in reflex pathways integral to maintaining stability.

## 5. Conclusion

This study demonstrates that elicited sensations via nerve cuff electrodes do not disrupt spinal reflex pathways during static postures with joint angles and loading conditions simulating early and mid-stance phases of the gait. This allows individuals with limb loss to benefit from sensory feedback perceived as originating from and co-located on their missing limbs without the risk of adversely affecting subconscious reflex processes crucial for maintaining stability. However, it would be challenging to directly extrapolate these findings from static postures to dynamic gait, since the sensory inputs from the periphery and descending cortical signals onto these spinal pathways are different during walking. Future work should quantify how EPS contributes to spinal reflex pathways during dynamic walking and other mobility tasks.

## Data availability statement

The raw data supporting the conclusions of this article will be made available by the authors, without undue reservation.

## Ethics statement

The studies involving humans were approved by Louis Stokes Cleveland Veterans Affairs Medical Center Institutional Review Board. The studies were conducted in accordance with the local legislation and institutional requirements. The participants provided their written informed consent to participate in this study. Written informed consent was obtained from the individual(s) for the publication of any potentially identifiable images or data included in this article.

## Author contributions

SL: Conceptualization, Data curation, Formal analysis, Investigation, Methodology, Software, Validation, Visualization, Writing – original draft, Writing – review & editing. RJT: Writing – review & editing, Supervision, Funding acquisition, Investigation, Project administration. HC: Writing – review & editing, Conceptualization, Supervision, Investigation, Project administration, Resources.
